# The role of indoleamine 2, 3 dioxygenase in regulating host immunity to leishmania infection

**DOI:** 10.1186/1423-0127-19-5

**Published:** 2012-01-09

**Authors:** Levi HC Makala

**Affiliations:** 1 Georgia Health Sciences University, Medical College of Georgia, Department of Pediatrics, Hematology/oncology Section, Augusta, Georgia 30912, USA

**Keywords:** **Leishimania major**, Indoleamine 2,3-dioxygenase, Mice, Leishmaniasis, Host Immunity, 1-methyl-d-tryptophan, plasmacytoid dendritic cell, regulatory T cell, draining lymph node

## Abstract

Pathogen persistence in immune-competent hosts represents an immunological paradox. Increasing evidence suggests that some pathogens, such as, *Leishmania major *(*L. major*) have evolved strategies and mechanisms that actively suppress host adaptive immunity. If this notion is correct conventional vaccination therapies may be ineffective in enhancing host immunity, unless natural processes that suppress host immunity are also targeted therapeutically. The key problem is that the basis of pathogen persistence in immune-competent individuals is unknown, despite decades of intense research. This fact, coupled with poor health care and a dearth of effective treatments means that these diseases will remain a scourge on humans unless a better understanding of why the immune system tolerates such infections emerges from research. Indoleamine 2,3-dioxygenase (IDO) has been shown to act as a molecular switch regulating host responses, and IDO inhibitor drugs shown to possess potential in enhancing host immunity to established leishmania infections. It is hoped that this review will help stimulate and help generate critical new knowledge pertaining to the IDO mechanism and how to exploit it to suppress T cell mediated immunity, thus offer an innovative approach to studying the basis of chronic leishmania infection in mice.

## Introduction

Chronic microbial infections, such as tuberculosis, AIDS, malaria and leishmaniasis, are a global health problem due to poor preventative health care in many developing countries, and lack of effective vaccines and drugs to prevent or clear chronic infections. Chronic infections pose an immunological paradox because pathogens persist in immune-competent hosts that, in principle, ought to mount effective immune responses leading to pathogen clearance. The fact that chronic infections persist under such circumstances suggests that pathogens exploit host immuno-regulatory mechanisms to protect themselves (and infected cells) by evading or actively suppressing host immunity. This perspective provides the rationale for identifying natural (host) immune-regulatory mechanisms exploited by pathogens and using this new knowledge to improve therapy.

This review summarizes new insights that are emerging from recent studies, focusing on why Leishmania persistence is achieved, and the other on DC subsets. These may well be inter-connected. It is a well-known fact that persistence is a hall mark of many parasitic infections. In this review, we attempt to uncover a novel mechanism by which Leishmania parasites circumvent effector T cell responses through the induction of indoleamine 2,3-dioxygenase (IDO) expression. Dendritic cell subsets (DC) are central in regulating immunity, often establishing immunologically privileged tissue micro environments through the induction of of T regulatory cells. One key concept is that only certain subsets of DCs seem competent to express IDO, at least at the level of functional immunoregulation. The factors that influence the development and tissue distribution of these IDO-competent DCs have yet to be determined. Unanswered issues include whether the different IDO-competent subsets arise from one or multiple lineages; and which signals during differentiation determine their ability to express IDO. One key mechanism that mediates many DC regulator functions is the production of the immunomodulatory enzyme IDO. For pathogens that cause chronic infection, for example leishmania, exploitation of DC and/or regulation of IDO presents a simple solution to persisting within a host. These two focuses are described later on in the text.

*Leishmania major *is an obligate intracellular and unicellular kinetoplastid protozoan flagellate that establishes itself within the phagolysosome of host immune competent cells, especially macrophages and dendritic cells (DCs). In 1903, W.B. Leishman and C. Donovan reported this new parasite at the turn of the century [1,2]. Ronald Ross christened the new genus Leishmania and the new species donovani in year 1903 [3]. *L. major *infection (Leishmaniasis) in mice is a widely used model of human infection that has yielded critical insights into the immunobiology of leishmaniasis [4-6]. Leishmaniasis as a parasitic disease manifests itself mainly in 3 clinical forms; visceral leishmaniasis (VL), cutaneous leishmaniasis (CL) and mucocutaneous leishmaniasis (MCL), of which VL is the most severe form of the disease. VL is lethal if untreated and spontaneous cure is extremely rare. Cutaneous leishmaniasis usually has milder course and often results into a self-healing of ulcers. Resolution of leishmanial infection is dependent on the coordinated interactions between components of cell mediated immune response, specifically the activation of targeted T-cell populations for appropriate cytokine production and activation of macrophages. *L. major *infection of B6 and BALB/c mouse strains drives predominantly T_H_1 and T_H_2 responses, respectively [7-10]. In murine model, the development of Thl response is associated with control of infection, and Th2 response is associated with disease progression. However, Th1 and Th2 dichotomy in the human system is not as distinct as in mice and the murine model does not strictly apply to human leishmaniasis.

Although T_H_1 responses are more effective than T_H_2 in controlling *L. major *infections, parasites are not eliminated completely by B6 or BALB/c hosts [11-13]. Thus *L. major *parasites persist in immunocompetent hosts, and mechanisms that explain *L. major *persistence are poorly defined hitherto. Suffia and colleagues demonstrated that Foxp3^+ ^regulatory T cells (Tregs) at *L. major *infection sites were parasite specific [14-16]. Tregs proliferated in response to *L. major *infected DCs and were restricted to sites of infection, and Treg survival depended on parasite persistence. These findings suggest that *L. major *parasites actively subvert host immune responses to suppress parasite-specific effector T cell responses. Unfortunately, counter-regulation induced in other settings of chronic inflammation promotes disease. Counter-regulation prevents excessive T cell immunity to innocuous substances encountered constitutively at mucosal surfaces during normal tissue functions. Chronic inflammation associated with other chronic infections and induced by some persistent pathogen infections is notoriously resistant to natural as well as vaccine-induced immunity. Resistance to T cell immunity is a significant barrier to successful immunotherapy to treat chronic infections such as HIV1, tuberculosis, and malaria. Moreover, most therapeutic vaccines (administered to patients with pre-existing conditions) have little effect on slowing disease progression, let alone stimulating pathogen clearance. Thus, local counter-regulatory processes that protect sites of infection are hurdles to effective immunotherapy. The well-established paradigm that mature DCs generated at sites of inflammation are T cell stimulatory is not consistent with these observations. A key question is whether mature DCs present antigens in a T cell stimulatory or T cell regulatory fashion in inflammatory settings where counter-regulation is predominant. Recent studies have shown that, indoleamine 2,3-dioxygenase (IDO) has drawn considerable attention as viable mechanism of immune regulation [17-20]. Other known mechanisms of immune regulation for Leishmania parasites are summarized in additional file 1 [reviewed in [21]]. Studies have demonstrated that IDO is an enzyme involved in the catabolism of the essential amino acid tryptophan, and the ratio of kynurenine, the main metabolite of tryptophan, to tryptophan (kyn/trp) can be used to reflect IDO activity. IDO is expressed mainly in antigen-presenting cells (APCs), i.e. at the initiation of the immune response. Its activation leads to a decrease in the tryptophan concentration in local microenvironments, thus suppressing the activation of surrounding T lymphocytes ('suppression by starvation') [18]. In a recent study [22] we reported that chronic infection of mice with the parasitic pathogen *Leishmania major *(*L. major*) induces localized T cell suppression due to induced expression of the enzyme indoleamine 2,3 dioxygenase (IDO). A key finding of our recent study [22] was that IDO inhibitor treatment reduced parasite burdens when administered to mice with established L. major infections, revealing a constitutive requirement for IDO to maintain local conditions that favor parasite persistence and pathogenesis and suggesting that 1-methyl-[D]-tryptophan (D-1MT) treatment may help reduce parasite burdens in patients with leishmaniasis. Our findings will provide new mechanistic insights into the role of IDO-expressing cells in creating immune privilege that blocks host anti-parasite immunity. This review focuses on the dichotomy of immune response against L. major infection and the basis of why pathogen persistence in immune-competent individuals is unknown, despite decades of intense research. A comprehensive understanding of sequences involved in the immune response to the parasite would help in designing prophylactic and therapeutic strategies against leishmaniasis and related infections.

## IDO: a natural T cell regulatory mechanism

Many study groups have shown that IDO is a conserved intracellular enzyme that degrades compounds containing indole rings, including the essential amino acid tryptophan [23-25]. In 1998, Mellor and Munn pioneered the discovery that IDO protected fetal allografts from potentially lethal maternal T cell immunity as shown by the fact that the IDO-specific inhibitor D-1MT induced fetal rejection when administered to pregnant mice [26]. The discovery of IDO as a modulator for the maintenance of fetomaternal immuno-privileged state has been heralded as a significant step in further defining the role of IDO in immunobiology. IDO is an IFN-inducible, intracellular enzyme that catalyzes the initial and rate-limiting step in the degradation of the essential amino acid, tryptophan. It has been suggested that IDO has the capacity to regulate the immune system via two discrete mechanisms; firstly the deprivation of tryptophan, which is essential for T cell proliferation and via the cytotoxic effects of tryptophan metabolites on T_H_1 cell survival. As we currently do not fully understand the remarkable immunosuppressive potency that is mediated by small numbers of IDO^+ ^DCs, it is probably wise to consider all potential mechanisms that might amplify the direct regulatory effects of IDO. Hypothetically, mechanisms by which suppression of adjacent T cells might occur are not limited to bystander suppression mediated by the IDO-expressing DC itself alone, because this may occur either through toxic metabolites, widespread local tryptophan depletion or IDO-induced regulatory cytokines. Macrophages, regulatory T or B cells might similarly be induced by IDO-responsive signalling pathways to secrete regulatory cytokines. Naive T cells (CD4^+ ^or CD8^+^) might be biased by IDO^+ ^DCs to adopt a regulatory phenotype. Any of these effects could then suppress nearby T cells responding to IDO^- ^antigen-presenting cells (APCs). The effect of IDO would therefore be to convert the local tissue microenvironment into a tolerizing milieu, even for antigens presented by other, normally immunogenic APCs. Additionally, this may involve a self-amplifying regulatory network involving interactions between IDO-competent DCs and regulatory T cells. The generation of regulatory T cells by IDO-expressing DCs could be a potent mechanism, as these regulatory T cells might in turn create other tolerogenic DCs through the induction of IDO expression by cytotoxic T lymphocyte antigen 4 (CTLA4) interactions. This could provide one possible molecular mechanism for epitope spreading and 'infectious' tolerance, as described in other systems [Reviewed in [27]]. Subsequent studies have shown that IDO activity regulates T cell responses in a range of inflammatory disease syndromes of clinical significance, including tumor growth and infectious, autoimmune & allergic diseases [27]. Thus, D-1MT treatment enhanced tumor-specific or pathogen (HIV)-specific T cell immunity in tumor and HIV-infection models [28-31]. Conversely, administering D-1MT to mice with experimental autoimmune and allergic disease syndromes increased disease severity and tissue pathology, and in some cases decreased survival. In addition several studies have shown that IDO inducers, genetically enhanced expression of IDO, or adoptive transfer of DCs induced to express IDO protects transplanted allogeneic tissues from attack by host T cells. These observations show that IDO is a natural immune-regulatory mechanism, which may be exploited by disease promoting agents, such as tumors and infectious pathogens, implying that the IDO mechanism may be manipulated for therapeutic benefit in patients with cancer and chronic infections.

## IDO-competent plasmacytoid DCs in mice and humans

IDO expression is tightly regulated and is induced in some cell types by infection and inflammation. Interferon (IFN) response elements in transcriptional promoters of mammalian IDO genes (ISRE, GAS) confer responsiveness to interferons. Though some non-hematopoietic cell types express IDO under inflammatory conditions, IDO expression is restricted to myeloid cells (macrophages, DCs) in hematopoietic cell lineages [32-34]. This review focuses on IDO expression in DC subsets because DCs are specialized to acquire, process and present antigens to T cells, leading to T cell activation. In recent published studies, Mellor and colleagues identified a specific subset of murine plasmacytoid DCs (CD19^+ ^pDCs) in spleen that express IDO selectively in response to inflammatory signals (mediated by IFN type 1, IFNα) induced by the synthetic reagents soluble CTLA4 (CTLA4-Ig) and DNA oligonucleotides containing un-methylated CpG motifs (CpGs) that ligate B7 (CD80/86) surface receptors, and intra-cellular Toll-Like Receptor-9 (TLR9), respectively [35-38]. These responses were highly specific for the small cohort of splenic CD19^+ ^pDCs, which rapidly produced IFNα after B7 and TLR9 ligation. IFNα was essential to induce IDO in CD19^+ ^pDCs via an auto/paracrine mechanism. Though CD19^+ ^pDCs are few in number (~10% of total splenic DCs) their IDO-dependent suppressive functions were dominant over the stimulatory functions of all other DC subsets, as shown by the fact that unfractionated DCs from B7 and TLR9 ligand treated mice exhibited potent T cell suppressive functions ex vivo.

In parallel studies, Munn and colleagues also identified IDO^+ ^pDC subsets in humans [39] and in tumor-draining LNs (TDLNs) in mice bearing melanomas [29,40]. In each case, IDO^+ ^pDCs exhibited potent T cell suppressive functions when cultured with T cells ex vivo. Collectively, these studies revealed that IDO expression was inducible in specific subsets of human and murine pDCs, and that the consequences of inducing IDO were that DCs acquired potent T cell suppressive functions.

## Infectious pathogens and IDO

Infectious pathogens provoke tissue inflammation, which causes some host cells (such as macrophages and NK cells) to release IFNs that induce IDO in some cells [41]. Previously, it was assumed that IDO induced by infections was a innate host defense mechanism that functioned by starving infected cells, and perhaps cancer cells, of tryptophan (an essential cell nutrient), which limited the spread of infections [42]. However the discovery that IDO has potent T cell regulatory effects when expressed by DCs suggests that this mechanism may be exploited to protect pathogens by suppressing host T cell mediated immunity directed at pathogens and infected cells. This notion provides the rationale and the need for future studies on the role of indoleamine 2, 3 dioxygenase in regulating host immunity to *Leishmania *infection. Studies on tumor-bearing mice provide additional support for the notion that the IDO mechanism is exploited by tumors to suppress anti-tumor immunity. In tumor-bearing mice abnormally high numbers of IDO^+ ^pDCs with potent T cell suppressive functions accumulated in tumor-draining LNs [29]. Thus, we hypothesize that pathogens, like tumors, may exploit the IDO mechanism to evade effective host T cell immunity potentially capable of completely eliminating chronic infections. If this hypothesis is verified, IDO-mediated suppression of pathogen-specific T cell immunity may be targeted therapeutically using IDO inhibitors to enhance effective T cell responses to pathogen infections. Importantly, IDO inhibitors are cheap to produce (e.g. D-1MT is an orphan drug) and should be relatively easy to administer to patients living in developing countries, two criteria emphasized by the Bill and Melinda Gates Foundation in promoting research on new approaches to treat chronic infections. Hence, testing the hypothesis above will be an important step in determining whether to proceed further with developing IDO inhibitors for potential therapeutic use in patients suffering from chronic infections.

## *Leishmania major *infection in mice: a model for chronic infection

In our recent studies [22], we observed IDO^+ ^cells in lymph nodes (LNs) draining sites of L. major infection in mice. We hypothesized that IDO^+ ^cells present in draining LNs create local suppressive microenvironments that facilitate *L. major *persistence by inhibiting T cell immunity to pathogen antigens. We selected the *L. major *infection in mice as the model system to test the hypothesis posed above for several reasons. First, the *L. major *infection model has been studied extensively by immunologists and was the original source of the T_H_1/T_H_2 'helper' T cell paradigm that provided key insights into alternative immune outcomes. Hence, *L. major *infection of B6 and BALB/c mouse strains drives predominantly T_H_1 and T_H_2 responses, respectively. T_H_1 responses are more effective in controlling *L. major *infections, but parasites are not eliminated completely by B6 or BALB/c hosts. Thus, unlike pathogens that cause acute infections (e.g. influenza virus), which are eventually eliminated, *L. major *parasites always persist in immune-competent hosts. Second, mechanisms that explain *L. major *persistence have not been defined, despite decades of research. Recently, Suffia et al., Belkaid et al, and Bertholet et al., reported the surprising findings that most FoxP3^+ ^CD4^+^CD25^+ ^regulatory T cells (Tregs) present at *L. major *infection sites in mice proliferated specifically in response to *L. major *infected DCs, were restricted to sites of infection, and that the survival of Tregs was strictly dependent on parasite persistence [43-45]. These findings suggested that host Tregs, which maintain self-tolerance, are subverted by *L. major *parasites to suppress host T cell immunity to parasite antigens. Third, Leishmaniasis threatens about 350 million men, women and children in 88 countries around the world. As many as 12 million people are believed to be currently infected by leishmaniasis, with about 1-2 million estimated new cases occurring every year worldwide [46,47]. Finally, drug regimens used against leishmaniasis (amphotericin B, pentamidine and pentostam) have limited efficacies and pose severe toxicity issues. Hence, there is an urgent need to develop less toxic and more efficacious drugs to target these chronic infectious pathogens.

This review cannot over emphasize the need to conduct studies to test the hypothesis that *L. major *infection in B6 mice induces IDO-competent pDCs to accumulate in LNs draining infection sites, which prevent effector T cell activation and activate resting, but functionally quiescent Tregs to acquire suppressor functions in this tissue microenvironment. The choice of B6 and BALB/c (DO11.10) mice for our recent study [22] was based on the need to use defined T cells from OT1 and OT2 T cell receptor transgenic (TCR Tg) with specificity for ovalbumin (OVA), as a means to test this hypothesis. A conceptual model of IDO-mediated activation and effector T cell suppression following *L. major *infection is summarized in additional file 2. The model depicts interactions between IDO+ DCs, Tregs and naïve T cells that drive suppressive and nonsuppressive outcomes under IDO-sufficient (+) and IDO-deficient (-) conditions in response to *L. major *infection. Induced IDO activity in DCs triggers cell stress responses, and blocks IL6 production by pDCs themselves, and by other cells (e.g. macrophages) capable of producing IL6. Under conditions of IDO ablation the same stimuli do not create suppression, and instead DCs stimulate naïve T cells, and express IL6, which converts Tregs to TH17 T cells or promotes TH17 differentiation from naïve CD4+ T cells.

New data [48,49] show that evidence for TH17 phenotype is related to Treg conversion under conditions of IDO ablation. The authors showed that IDO activates Tregs and blocks their conversion into TH17-like T cells supporting the hypothesis that IDO dominantly controls the functional status of Tregs in response to inflammatory stimuli in physiological setting. Links between L. major and activation of regulatory T cells have been described though molecular mechanisms that explain these links have not been described. Induced IDO expression is strongly linked to activation of Tregs in response to tumor growth and TLR9 ligand treatment in mice, forming the basis of the guiding hypothesis we put forward. The hypothesis that IDO inhibits natural (T cell mediated) immunity is novel, though not universally accepted. The related notion that IDO contributes to L. major persistence represents a novel paradigm. However, even the notion that tumors or infectious pathogens exploit the natural immunoregulatory IDO mechanism to suppress T cell immunity does not yet enjoy universal acceptance in the field of immunology. It would be interesting to perform studies to test this hypothesis, by assessing the effects of IDO-regulated IL17 production on Tregs and TH17 T cells, using the Treg specific marker, Foxp3. The use of Foxp3GFPKO mice that express a Foxp3-GFP fusion protein [50] may likely clear this controversy. This hypothesis can be addressed further by determining the activation status of Tregs using MLRs and cytokine profile analysis in the infected relevant mice treated with oral D-1MT as well as IDO1-KO mice.

## Innovative Immunotherapy: Destruction of local Immune privilege

The global diseases tuberculosis, AIDS, malaria and leishmaniasis are caused by chronic pathogenic infections that are not cleared by natural immunity. Typically treatments slow disease progression rather than inducing pathogen clearance. Attempts to develop effective therapeutic vaccines to clear chronic infections have consistently failed. Even the search for effective prophylactic vaccines is frustrating, as evidenced by the recent failure of Merck's Step vaccine to prevent new HIV infections in 'at risk' individuals [51-53]. New perspectives leading to innovative treatments are urgently needed to address the global problem of chronic infections. From an immunologic perspective pathogen persistence in individuals with competent immune systems contradicts the fundamental tenet of immunity to non-self and tolerance to self, prompting the question; why do pathogen (non-self) antigens fail to stimulate effective immunity that leads to pathogen clearance? The answer to this question may lie in the fact that some pathogens evolved the ability to create local immune privilege where immunity to non-self antigens is actively suppressed. This novel perspective explains why traditional vaccine approaches are ineffective, even when vaccines stimulate systemic immunity, and implies that natural immune regulatory mechanisms protect pathogens and infected cells from host immunity at local sites of infection. Most importantly this perspective suggests that novel therapies that destroy immune privilege will be effective methods to eliminate persistent pathogen infections. Acquired immune privilege is defined as a set of local mechanisms that suppress the mature lymphocyte responses to antigens encountered in peripheral tissues [54]; for example, acute *L. major *infection elicits local IL10 production [55,56] which limits collateral damage during initial infection, but is also immunosuppressive and allows the pathogen to persist [57].

Various groups have been studying a specific immune regulatory mechanism that involves expression of the enzyme indoleamine 2,3 dioxygenase (IDO) by particular cell types such as certain subsets of dendritic cells (DCs) [58-63]. IDO is induced by inflammation and by pathogens that cause all the infectious diseases mentioned above. We hypothesize that local IDO expression creates immune privilege at local sites of persistent infection. The specific goal of the future studies should be to test the prediction that IDO blockade coupled with chemotherapy/vaccine therapy to destroy established immune privilege, and block its re-establishment will lead to pathogen clearance. The rationale for this innovative approach is based on evidence that IDO inhibitors and certain chemotherapeutic agents synergize to drive more effective immune-mediated tumor clearance in mice [64-66]; indeed Phase I clinical trials in cancer patients to test the efficacy of the IDO inhibitor 1-methyl-[D]-tryptophan (D-1MT) as a vaccine adjuvant are underway at two US medical centers. In recent studies [22] we discovered that cutaneous *L. major *infection in mice induced DCs in lymph nodes draining infected skin to express IDO, just as in lymph nodes draining sites of melanoma growth [66]. These findings suggest that *L. major *infection creates immune privilege by inducing DCs in local inflamed lymph nodes to express IDO. Hence the specific goal of future studies should be targeted to treating *L. major *infected B6 and BALB/c mice with D-1MT and autoclaved leishmania vaccine (ALM), and monitor the potential therapeutic effects of combined immuno-chemotherapy on reducing parasite loads and affecting parasite clearance. The speedy execution and completion of studies targeted on therapeutic potential and or immunochemotherapy will provide critical mechanistic insights into the role of IDO-expressing cells in creating immune privilege that blocks host anti-parasite immunity.

## Limitations of IDO and Leishmania Research

Overall, mechanisms that explain *L. major *persistence have not been defined, despite decades of research. No effective vaccine is yet available against this parasite and its control relies primarily on chemotherapy. Since the discovery of the pentavalent antimonials, (the first drug for leishmaniasis treatment), [67], hitherto, the search for lead molecules with anti-leishmanial activity, without toxic effects, and able to overcome the emergence of drug resistant strains, still remains viable limitation. Poor health care and a deficiency of effective treatments and cures means that Leishmania will remain a scourge on humans unless a better understanding of why the immune system tolerates such infections emerges from research. Clearly, the identification and an in-depth understanding of the type(s) of cells in which the parasites persist, and the elucidation of the mechanisms that are essential for maintaining a stable host-parasite relationship, will be major tasks for the future. Although parasite persistence always bears the risk of later reactivation and reappearance of the disease, it remains uncertain to date whether sterile immunity would be advantageous to the host because parasite elimination might also blunt T-cell memory and thereby enable reinfection of the host. However, even without the goal to find new strategies for the killing of hidden parasites *in vivo*, studies on the mechanisms of parasite evasion will certainly enrich our understanding of the anti-parasite immune defense.

## Conclusions

As summarized above, various study groups have identified a small subset of splenic IDO- competent pDCs that are capable of influencing immune outcomes greatly by expressing IDO and acquiring potent T cell suppressive functions with a capacity to block the stimulatory properties of T cells [22,66]. These findings showed that IDO induced by L. major infection attenuated innate and adaptive immune responses. Thus, IDO may act as a molecular switch regulating host responses, and IDO inhibitor drugs are a potential new approach to enhance host immunity to established leishmania infections.

The main goal of future research should be to generate new knowledge to exploit the IDO mechanism for optimal therapeutic gain in patients with leishmaniasis and other similar infectious pathogens. Immunocompetent humans eventually heal L. major lesions, inducing a life-long powerful immunity. The only efficacious vaccine against leishmaniasis is Leishmanization, the process of inoculating individuals with live parasites. It is thought and felt that maintenance of immunity to Leishmania infection requires the presence of a chronic infection. Before IDO inhibitors are utilized to treat leishmaniasis. It will be necessary to determine how suppression of IDO will influence parasite persistence and the induction of protective immunity. Indeed, to this end studies must be carried out to investigate the effects of inducing and ablating IDO in mice during immunization against leishmaniasis. It is felt that such investigative research will generate critical new knowledge pertaining to the IDO mechanism and how to exploit it to suppress T cell mediated immunity.

## List of abbreviations

(IDO): indoleamine 2,3-dioxygenase; (1M(D)T): 1-methyl-d-tryptophan; (MLR): mixed leukocyte reaction; (pDC): plasmacytoid DC; (Treg): regulatory T cell; (TCR): T cell receptor; (TDLN): tumor-draining LN; (dLN): draining inguinal LN.

## Competing interests

The author declares that they have no competing interests.

## Authors' contributions

The author read and approved the final manuscript

## Supplementary Material

Additional file 1 **Other known mechanisms of immune regulation for Leishmania parasites**. Click here for file

Additional File 2 **Conceptual model of IDO-mediated activation and effector T cell suppression following *L. major *infection**. The model depicts interactions between IDO+ DCs, Tregs and naïve T cells that drive suppressive and non-suppressive outcomes under IDO-sufficient (+) and IDO-deficient (-) conditions in response to *L. major *infection. Induced IDO activity in DC's triggers cell stress responses and blocks IL6 production by pDCs themselves, and by other cells (e.g. macrophages), capable of producing IL6. Under conditions of IDO ablation the same stimuli do not create suppression, and instead DCs stimulate naïve T cells, and express IL6, which converts Tregs to TH17 T cells or promotes TH17 differentiation from naïve CD4+ T cells. Click here for file

## References

[B1] Leishman WB "On the possibility of the occurrence of trypanomiasis in India" The British Medical Journal 1903 7950960

[B2] Donovan C "Memoranda: On the possibility of the occurence of trypanomiasis in India." The British Medical Journal 1903

[B3] Ross R "Further notes on Leishman's bodies" Ibid 1903 ii 1401 10.1136/bmj.2.2239.1401 PMC2514909 20761210

[B4] Peters N Sacks D Immune privilege in sites of chronic infection: Leishmania and regulatory T cells Immunolo Rev 2006 213 159 179 10.1111/j.1600-065X.2006.00432.x 16972903

[B5] Liese J Schleicher U Bogdan C The innate immune response against Leishmania parasites Immunobiology 2008 213 377 387 10.1016/j.imbio.2007.12.005 18406382

[B6] Sharma U Singh S Immunobiology of leishmaniasis Indian J Exp Biol 2009 47 412 423 19634705

[B7] Moll H Rollinghoff M Resistance to murine cutaneous leishmaniasis is mediated by TH1 cells, but disease-promoting CD41 cells are different from TH2 cells Eur J Immunol 1990 20 2067 2074 10.1002/eji.1830200927 1976523

[B8] Lohoff M Sommer F Solbach W Rollinghoff M Coexistence of antigen-specific TH1 and TH2 cells in genetically susceptible BALB/c mice infected with Leishmania major Immunobiology 1989 179 412 421 10.1016/S0171-2985(89)80045-2 2575598

[B9] Locksley RM Heinzel FP Holaday BJ Mutha SS Reiner SL Sadick MD Induction of Th1 and Th2 CD41 subsets during murine Leishmania major infection Res Immunol 1991 142 28 32 10.1016/0923-2494(91)90007-6 1829257

[B10] Scott P IFN-gamma modulates the early development of Th1 and Th2 responses in a murine model of cutaneous leishmaniasis J Immunol 1991 147 3149 55 1833466

[B11] Scott P Natovitz P Coffman RL Pearce E Sher A Immunoregulation of cutaneous leishmaniasis. T cell lines that transfer protective immunity or exacerbation belong to different T helper subsets and respond to distinct parasite antigens J Exp Med 1988 168 1675 84 10.1084/jem.168.5.1675 2903212 PMC2189120

[B12] Holaday BJ Sadick MD Wang ZE Reiner SL Heinzel FP Parslow TG Locksley RM Reconstitution of Leishmania immunity in severe combined immunodeficient mice using Th1- and Th2-like cell lines J Immunol 1991 147 1653 8 1831830

[B13] Reiner SL Locksley RM The regulation of immunity to Leishmania major Annu Rev Immunol 1995 13 151 77 10.1146/annurev.iy.13.040195.001055 7612219

[B14] Suffia IJ Reckling SK Piccirillo CA Goldszmid RS Belkaid Y Infected site-restricted Foxp31 natural regulatory T cells are specific for microbial antigens J Exp Med 2006 203 777 88 10.1084/jem.20052056 16533885 PMC2118233

[B15] Belkaid Y Tarbell K Regulatory T cells in the control of hostmicroorganism interactions (*) Annu Rev Immunol 2009 27 551 89 10.1146/annurev.immunol.021908.132723 19302048

[B16] Belkaid Y Tarbell KV Arming Treg cells at the inflammatory site Immunity 2009 30 322 3 10.1016/j.immuni.2009.03.004 19303386

[B17] Friberg M Jennings R Alsarraj M Dessureault S Cantor A Extermann M Mellor AL Munn DH Antonia SJ Indoleamine 2,3-dioxygenase contributes to tumor cell evasion of T cell-mediated rejection International Journal of Cancer 2002 101 151 155 10.1002/ijc.10645 12209992

[B18] Munn DH Sharma MD Hou D Baban B Lee J Antonia SJ Messina JL Chandler P Koni PA Mellor AL Expression of indoleamine 2,3-dioxygenase by plasmacytoid dendritic cells in tumor-draining lymph nodes J Clin Invest 2004 114 280 290 15254595 10.1172/JCI21583 PMC449750

[B19] Potula R Poluektova L Knipe B Chrastil J Heilman D Dou H Takikawa O Munn DH Gendelman HE Persidsky Y Inhibition of indoleamine 2,3-dioxygenase (IDO) enhances elimination of virus-infected macrophages in an animal model of HIV-1 encephalitis Blood 2005 106 2382 2390 10.1182/blood-2005-04-1403 15961516 PMC1895260

[B20] Munn DH Mellor AL Indoleamine 2,3-dioxygenase and tumor-induced tolerance J Clin Invest 2007 117 1147 1154 10.1172/JCI31178 17476344 PMC1857253

[B21] Bogdan C Röllinghoff M How do Protozoan Parasites Survive inside Macrophages? Parasitology Today 1999 15 1 22 28 10.1016/S0169-4758(98)01362-3 10234174

[B22] Makala LH Baban B Lemos H El-Awady AR Chandler PR Hou DY Munn DH Mellor AL Leishmania major Attenuates Host Immunity by Stimulating Local Indoleamine 2,3-Dioxygenase Expression J Infect Dis 2011 203 715 25 10.1093/infdis/jiq095 21282196 PMC3072725

[B23] Mellor AL Munn DH Tryptophan catabolism and T-cell tolerance: immunosuppression by starvation? Immunol Today 1999 20 469 473 10.1016/S0167-5699(99)01520-0 10500295

[B24] Sugimoto H Oda S Otsuki T Hino T Yoshida T Shiro Y Crystal structure of human indoleamine 2,3-dioxygenase: catalytic mechanism of O2 incorporation by a heme-containing dioxygenase Proc Natl Acad Sci USA 2006 103 2611 2616 10.1073/pnas.0508996103 16477023 PMC1413787

[B25] Taylor MW Feng GS Relationship between interferon-gamma, indoleamine 2,3-dioxygenase, and tryptophan catabolism FASEB J 1991 5 2516 2522 1907934

[B26] Munn DH Zhou M Attwood JT Bondarev I Conway SJ Marshall B Brown C Mellor AL Prevention of allogeneic fetal rejection by tryptophan catabolism Science 1998 281 1191 1193 9712583 10.1126/science.281.5380.1191

[B27] Mellor AL Munn DH IDO expression in dendritic cells: Tolerance and tryptophan catabolism Nat Rev Immunol 2004 4 762 774 10.1038/nri1457 15459668

[B28] Friberg M Jennings R Alsarraj M Dessureault S Cantor A Extermann M Mellor AL Munn DH Antonia SJ Indoleamine 2,3-dioxygenase contributes to tumor cell evasion of T cell-mediated rejection International Journal of Cancer 2002 101 151 155 10.1002/ijc.10645 12209992

[B29] Munn DH Sharma MD Hou D Baban B Lee J Antonia SJ Messina JL Chandler P Koni PA Mellor AL Expression of indoleamine 2,3-dioxygenase by plasmacytoid dendritic cells in tumor-draining lymph nodes J Clin Invest 2004 114 280 290 15254595 10.1172/JCI21583 PMC449750

[B30] Potula R Poluektova L Knipe B Chrastil J Heilman D Dou H Takikawa O Munn DH Gendelman HE Persidsky Y Inhibition of indoleamine 2,3-dioxygenase (IDO) enhances elimination of virus-infected macrophages in an animal model of HIV-1 encephalitis Blood 2005 106 2382 2390 10.1182/blood-2005-04-1403 15961516 PMC1895260

[B31] Munn DH Mellor AL Indoleamine 2,3-dioxygenase and tumor-induced tolerance J Clin Invest 2007 117 1147 1154 10.1172/JCI31178 17476344 PMC1857253

[B32] Munn DH Sharma MD Baban B Harding HP Zhang Y Ron D Mellor AL GCN2 kinase in T cells mediates proliferative arrest and anergy induction in response to indoleamine 2,3-dioxygenase Immunity 2005 22 633 642 10.1016/j.immuni.2005.03.013 15894280

[B33] Sharma MD Hou DY Liu Y Koni PA Metz R Chandler P Mellor AL He Y Munn DH Indoleamine 2,3-dioxygenase controls conversion of Foxp3+ Tregs to TH17-like cells in tumor-draining lymph nodes Blood 2009 113 6102 6111 10.1182/blood-2008-12-195354 19366986 PMC2699232

[B34] Mellor AL Munn DH Creating immune privilege: active local suppression that benefits friends, but protects foes Nat Rev Immunol 2008 8 74 80 10.1038/nri2233 18064049

[B35] Mellor AL Baban B Chandler P Marshall B Jhaver K Hansen A Koni PA Iwashima M Munn DH Cutting edge: induced indoleamine 2,3 dioxygenase expression in dendritic cell subsets suppresses T cell clonal expansion J Immunol 2003 171 1652 1655 12902462 10.4049/jimmunol.171.4.1652

[B36] Mellor AL Chandler PR Baban B Hansen AM Marshall B Pihkala J Waldmann H Cobbold SP Adams E Munn DH Specific subsets of murine dendritic cells acquire potent T cell regulatory functions following CTLA4-mediated induction of indoleamine 2,3 dioxygenase Int Immunol 2004 16 1391 1401 10.1093/intimm/dxh140 15351783

[B37] Baban B Hansen AM Chandler PR Manlapat A Bingaman A Kahler DJ Munn DH Mellor AL A minor population of splenic dendritic cells expressing CD19 mediates IDO-dependent T cell suppression via type I IFN signaling following B7 ligation Int Immunol 2005 17 909 919 10.1093/intimm/dxh271 15967784

[B38] Mellor AL Baban B Chandler PR Manlapat A Kahler DJ Munn DH Cutting Edge: CpG Oligonucleotides Induce Splenic CD19+ Dendritic Cells to Acquire Potent Indoleamine 2,3-Dioxygenase-Dependent T Cell Regulatory Functions via IFN Type 1 Signaling J Immunol 2005 175 5601 5605 16237046 10.4049/jimmunol.175.9.5601

[B39] Munn DH Sharma MD Lee JR Jhaver KG Johnson TS Keskin DB Marshall B Chandler P Antonia SJ Burgess R Slingluff CL Mellor AL Potential regulatory function of human dendritic cells expressing indoleamine 2,3-dioxygenase Science 2002 297 1867 1870 10.1126/science.1073514 12228717

[B40] Munn DH Sharma MD Baban B Harding HP Zhang Y Ron D Mellor AL GCN2 Kinase in T Cells Mediates Proliferative Arrest and Anergy Induction in Response to Indoleamine 2,3-Dioxygenase Immunity 2005 22 1 10 10.1016/j.immuni.2005.01.001 15894280

[B41] Guillonneau C Mintern JD Hubert FX Hurt AC Besra GS Porcelli S Barr IG Doherty PC Godfrey DI Turner SJ Combined NKT cell activation and influenza virus vaccination boosts memory CTL generation and protective immunity Proc Natl Acad Sci USA 2009 106 3330 3335 10.1073/pnas.0813309106 19211791 PMC2651345

[B42] Taylor MW Feng G Relationship between interferon-g, indoleamine 2,3-dioxygenase, and tryptophan catabolism FASEB Journal 1991 5 2516 2522 1907934

[B43] Suffia IJ Reckling SK Piccirillo CA Goldszmid RS Belkaid Y Infected site-restricted Foxp3+ natural regulatory T cells are specific for microbial antigens J Exp Med 2006 203 777 788 10.1084/jem.20052056 16533885 PMC2118233

[B44] Belkaid Y Blank RB Suffia I Natural regulatory T cells and parasites: a common quest for host homeostasis Immunol Rev 2006 212 287 300 10.1111/j.0105-2896.2006.00409.x 16903921

[B45] Bertholet S Debrabant A Afrin F Caler E Mendez S Tabbara KS Belkaid Y Sacks DL Antigen requirements for efficient priming of CD8+ T cells by Leishmania major-infected dendritic cells Infect Immun 2005 73 6620 6628 10.1128/IAI.73.10.6620-6628.2005 16177338 PMC1230980

[B46] Qi H Ji J Wanasen N Soong L Enhanced replication of Leishmania amazonensis amastigotes in gamma interferon-stimulated murine macrophages: implications for the pathogenesis of cutaneous leishmaniasis Infect Immun 2004 72 988 995 10.1128/IAI.72.2.988-995.2004 14742545 PMC321581

[B47] WHO Programme for the surveillance and control of leishmaniasis 2001 http://www.who.int/emc/diseases/leish/leishmaniasis.pdf. (Internet publication)

[B48] Baban B Chandler PR Sharma MD Pihkala J Koni PA Munn DH Mellor AL IDO activates regulatory T cells and blocks their conversion into Th17-like T cells J Immunol 2009 183 2475 2483 10.4049/jimmunol.0900986 19635913 PMC3677163

[B49] Sharma MD Baban B Chandler PR Hou DY Singh N Yagita H Azuma M Blazar BR Mellor AL Munn DH Plasmacytoid dendritic cells from mouse tumor-draining lymph nodes activate mature Tregs via indoleamine 2,3-dioxygenase J Clin Invest 2007 117 1 13 10.1172/JCI31911 PMC1940240 17710230

[B50] Fontenot JD Rasmussen JP Williams LM Dooley JL Farr AG Rudensky AY Regulatory T cell lineage specification by the forkhead transcriptionfactor foxp3 Immunity 2005 22 329 341 10.1016/j.immuni.2005.01.016 15780990

[B51] HIV vaccine failure prompts Merck to halt trial Nature 2007 449 390 17898737 10.1038/449390c

[B52] Robb ML Failure of the Merck HIV vaccine: an uncertain step forward Lancet 2008 372 1857 1858 10.1016/S0140-6736(08)61593-7 19012958

[B53] Watkins DI Burton DR Kallas EG Moore JP Koff WC Nonhuman primate models and the failure of the Merck HIV-1 vaccine in humans Nat Med 2008 14 617 621 10.1038/nm.f.1759 18535579 PMC3697853

[B54] Mellor AL Munn DH Creating immune privilege: active local suppression that benefits friends, but protects foes Nat Rev Immunol 2008 8 74 80 10.1038/nri2233 18064049

[B55] Peters N Sacks D Immune privilege in sites of chronic infection: Leishmania and regulatory T cells Immunol Rev 2006 213 159 179 10.1111/j.1600-065X.2006.00432.x 16972903

[B56] Trinchieri G Interleukin-10 production by effector T cells: Th1 cells show self control J Exp Med 2007 204 239 243 10.1084/jem.20070104 17296790 PMC2118719

[B57] Belkaid Y Hoffmann KF Mendez S Kamhawi S Udey MC Wynn TA Sacks DL The role of interleukin (IL)-10 in the persistence of Leishmania major in the skin after healing and the therapeutic potential of anti-IL-10 receptor antibody for sterile cure J Exp Med 2001 194 1497 1506 10.1084/jem.194.10.1497 11714756 PMC2193677

[B58] Mellor AL Munn DH IDO expression by dendritic cells: tolerance and tryptophan catabolism Nat Rev Immunol 2004 4 762 774 10.1038/nri1457 15459668

[B59] Mellor AL Chandler P Baban B Hansen AM Marshall B Pihkala J Waldmann H Cobbold S Adams E Munn DH Specific subsets of murine dendritic cells acquire potent T cell regulatory functions following CTLA4-mediated induction of indoleamine 2,3 dioxygenase Int Immunol 2004 16 1391 1401 10.1093/intimm/dxh140 15351783

[B60] Munn DH Sharma MD Hou D Baban B Lee JR Antonia SJ Messina JL Chandler P Koni PA L Mellor A Expression of indoleamine 2,3-dioxygenase by plasmacytoid dendritic cells in tumor-draining lymph nodes J Clin Invest 2004 114 280 290 15254595 10.1172/JCI21583 PMC449750

[B61] Baban B Hansen AM Chandler PR Manlapat A Bingaman A Kahler DJ Munn DH Mellor AL A minor population of splenic dendritic cells expressing CD19 mediates IDO-dependent T cell suppression via type I IFN signaling following B7 ligation Int Immunol 2005 17 909 919 10.1093/intimm/dxh271 15967784

[B62] Hou DY Muller AJ Sharma MD DuHadaway J Banerjee T Johnson M Mellor AL Prendergast GC Munn DH Inhibition of indoleamine 2,3-dioxygenase in dendritic cells by stereoisomers of 1-methyl-tryptophan correlates with antitumor responses Cancer Res 2007 67 792 801 10.1158/0008-5472.CAN-06-2925 17234791

[B63] Muller AJ DuHadaway JB Donover PS Sutanto-Ward E Prendergast GC Inhibition of indoleamine 2,3-dioxygenase, an immunoregulatory target of the cancer suppression gene Bin1, potentiates cancer chemotherapy Nat Med 2005 11 312 319 10.1038/nm1196 15711557

[B64] Muller AJ Sharma MD Chandler PR Duhadaway JB Everhart ME Johnson BA Kahler DJ Pihkala J Soler AP Munn DH Prendergast GC Mellor AL Chronic inflammation that facilitates tumor progression creates local immune suppression by inducing indoleamine 2,3 dioxygenase Proc Natl Acad Sci USA 2008 105 17073 17078 10.1073/pnas.0806173105 18952840 PMC2579380

[B65] Sharma MD Baban B Chandler P Hou DY Singh N Yagita H Azuma M Blazar BR Mellor AL Munn DH Plasmacytoid dendritic cells from mouse tumor-draining lymph nodes directly activate mature Tregs via indoleamine 2,3-dioxygenase J Clin Invest 2007 117 2570 2582 10.1172/JCI31911 17710230 PMC1940240

[B66] Andersen MH A potential role of indoleamine 2,3-dioxygenase-specific T cells in leishmania vaccination. (Comment on J Infect Dis. 2011 Mar 1;203(5):715-25) J Infect Dis 2011 204 488 489 10.1093/infdis/jir274 21742850 PMC3132142

[B67] No Authors The first drug for leishmaniasis treatment IJMR 1922 10 492

