# Supplementary material for: The role of indoleamine 2, 3 dioxygenase in regulating host immunity to leishmania infection
Source: J Biomed Sci. 2012 Jan 9;19(1):5. doi: 10.1186/1423-0127-19-5 (PMC3295648; doi:10.1186/1423-0127-19-5)
Supplement: Additional file 1 — Other known mechanisms of immune regulation for Leishmania parasites. [file 1423-0127-19-5-S1.DOCX]

| Table 1. Strategies and mechanisms of survival of Leishmania parasites in immune competent cells (APC’s) | | | |
| --- | --- | --- | --- |
| **Strategy** | **Mechanism** | **Leishmania species** | **References** |
| Tendency to retreat into a safe intracellular compartments | Lack of fusion between parasitophorous  vacuoles and lysosomes  Inhibition of phagolysosomal proteases | *Leishmania donovani*  *Leishmania* spp | 1, 2  1, 3, 4 |
| Tendency to suppress the synthesis of microbicidal radicals | Inhibition of iNOS expression or activity  Inhibition of oxidative burst | *L. major* (GIPL, phosphoglycan)  *Leishmania* spp (LPG) | 3, 5,  1, 3 |
| Ability to modulate of APC’s apoptosis | Enhanced APC’s survival after infection | *L. donovani* | 6 |
| Ability to modulate of APC’s cytokine production | Induction of cytokines inhibiting/deactivating  APC (e.g. IL-10, TGF-β)  Suppression or lack of induction of activating cytokines (e.g. IL-12, IL-17) | *Leishmania* spp,  *Leishmania* spp, | 7–15  13, 14, 22 |
| Ability to inhibit antigen presentation and T-cell stimulation | Reduced MHC class II expression by APC’s  Reduced expression of co-stimulatory  molecules by APC’s  Inhibition of antigen processing/peptide  loading, sequestration of antigen from  presentation  Induction of IDO inhibiting APC | *L. donovani*, *L. amazonensis*  *L. donovani*  *L. major*, *L. donovani*, *L. amazonensis*  *L. Mexicana*  *L. major* | 3, 16, 17  3, 16, 17  3, 16, 17, 22 |
|  |  |  |  |

Abbreviations: GIPL, glycoinositolphospholipids; IL, interleukin; iNOS, inducible nitric oxide synthase (NOS2); LPG, lipophosphoglycan; MHC, major histocompatibility complex; TGF-β, transforming growth factor β; APC’s, antigen presenting cells; IDO, indoleamine 2,3-dioxygenase

**References for table 1**(Reference #22 is reflected in the main text as well)

1. Mauël J: **Intracellular survival of protozoan parasites with special reference to *Leishmania* spp, *Toxoplasma gondii*, and *Trypanosoma cruzi***. *Adv. Parasitol.* 1996, **38:**1–51.
2. Desjardins M and Descoteaux A: **Inhibition of phagolysosomal biogenesis by the *Leishmania* lipophosphoglycan,** *J. Exp. Med.* 1997, **185:**2061–2068.
3. Bogdan C and Röllinghoff M: **The immune response to *Leishmania*: mechanisms of parasite control and evasion**. *Int. J. Parasitol.* 1998, **28:**121–134.
4. Joshi PB, [Sacks DL](http://www.ncbi.nlm.nih.gov/pubmed?term=%22Sacks%20DL%22%5BAuthor%5D), [Modi G](http://www.ncbi.nlm.nih.gov/pubmed?term=%22Modi%20G%22%5BAuthor%5D), [McMaster WR](http://www.ncbi.nlm.nih.gov/pubmed?term=%22McMaster%20WR%22%5BAuthor%5D): **Targeted deletion of Leishmania major genes encoding developmental stage-specific leishmaniolysin (gp63)**. *Mol. Microbiol.* 1998, **27:**519–530.
5. Plasman N [Metz G](http://www.ncbi.nlm.nih.gov/pubmed?term=%22Metz%20G%22%5BAuthor%5D), [Vray B](http://www.ncbi.nlm.nih.gov/pubmed?term=%22Vray%20B%22%5BAuthor%5D). **Interferon-γ-activated immature macrophages exhibit a high *Trypanosoma cruzi* infection rate associated with a low production of both nitric oxide and tumor necrosis factor alpha**. *Parasitol. Res.* 1994, **80:**554–558.
6. Moore KJ and Matlashewski G: **Intracellular infection by *Leishmania donovani* inhibits macrophage apoptosis**. *J. Immunol.* 1994, **152:**2930–2937.
7. Bogdan C and Nathan C: **Modulation of macrophage function by transforming growth factor-β, interleukin 4 and interleukin 10**. Ann. New York Acad. Sci. 1993, **685:**713–739.
8. Barral A, [Barral-Netto M](http://www.ncbi.nlm.nih.gov/pubmed?term=%22Barral-Netto%20M%22%5BAuthor%5D), [Yong EC](http://www.ncbi.nlm.nih.gov/pubmed?term=%22Yong%20EC%22%5BAuthor%5D), [Brownell CE](http://www.ncbi.nlm.nih.gov/pubmed?term=%22Brownell%20CE%22%5BAuthor%5D), [Twardzik DR](http://www.ncbi.nlm.nih.gov/pubmed?term=%22Twardzik%20DR%22%5BAuthor%5D), [Reed SG](http://www.ncbi.nlm.nih.gov/pubmed?term=%22Reed%20SG%22%5BAuthor%5D): **Transforming growth factor β as a virulence mechanism for *Leishmania braziliensis*,** *Proc. Natl. Acad. Sci. U. S. A.* 1993, **90:**3442–3446.
9. Stenger S [Thüring H](http://www.ncbi.nlm.nih.gov/pubmed?term=%22Th%C3%BCring%20H%22%5BAuthor%5D), [Röllinghoff M](http://www.ncbi.nlm.nih.gov/pubmed?term=%22R%C3%B6llinghoff%20M%22%5BAuthor%5D), [Bogdan C](http://www.ncbi.nlm.nih.gov/pubmed?term=%22Bogdan%20C%22%5BAuthor%5D): **Tissue expression of inducible nitric oxide synthase is closely associated with resistance to *Leishmania major***, *J. Exp. Med.* 1994, **180:**783–793.
10. Rodrigues V [Santana da Silva J](http://www.ncbi.nlm.nih.gov/pubmed?term=%22Santana%20da%20Silva%20J%22%5BAuthor%5D), [Campos-Neto A](http://www.ncbi.nlm.nih.gov/pubmed?term=%22Campos-Neto%20A%22%5BAuthor%5D): **Transforming growth factor-β and immunosuppression in experimental visceral leishmaniasis.** *Infect. Immun.* 1998 **66:**1233–1236.
11. Silva JS, [Twardzik DR](http://www.ncbi.nlm.nih.gov/pubmed?term=%22Twardzik%20DR%22%5BAuthor%5D), [Reed SG](http://www.ncbi.nlm.nih.gov/pubmed?term=%22Reed%20SG%22%5BAuthor%5D): **Regulation of *Trypanosoma cruzi* infections *in vitro* and *in vivo* by transforming growth factor β (TGF-β).** *J. Exp. Med.* 1991, **174:**539–545.
12. Bermudez LE Covaro G, Remington J: **Infection of murine macrophages with *Toxoplasma gondii* is associated with the release of transforming growth factor β and downregulation of expression of tumor necrosis factor receptors.** *Infect. Immun.* 1993, **61:**4126–4130.
13. Carrera L, [Gazzinelli RT](http://www.ncbi.nlm.nih.gov/pubmed?term=%22Gazzinelli%20RT%22%5BAuthor%5D), [Badolato R](http://www.ncbi.nlm.nih.gov/pubmed?term=%22Badolato%20R%22%5BAuthor%5D), [Hieny S](http://www.ncbi.nlm.nih.gov/pubmed?term=%22Hieny%20S%22%5BAuthor%5D), [Muller W](http://www.ncbi.nlm.nih.gov/pubmed?term=%22Muller%20W%22%5BAuthor%5D), [Kuhn R](http://www.ncbi.nlm.nih.gov/pubmed?term=%22Kuhn%20R%22%5BAuthor%5D), [Sacks DL](http://www.ncbi.nlm.nih.gov/pubmed?term=%22Sacks%20DL%22%5BAuthor%5D)*:* ***Leishmania* promastigotes selectively inhibit interleukin-12 induction in bone marrow-derived macrophages from susceptible and resistant mice.** *J. Exp. Med.* 1996, **183:**515–526.
14. Sartori A, [Oliveira MA](http://www.ncbi.nlm.nih.gov/pubmed?term=%22Oliveira%20MA%22%5BAuthor%5D), [Scott P](http://www.ncbi.nlm.nih.gov/pubmed?term=%22Scott%20P%22%5BAuthor%5D), [Trinchieri G](http://www.ncbi.nlm.nih.gov/pubmed?term=%22Trinchieri%20G%22%5BAuthor%5D): **Metacyclogenesis modulates the ability of *Leishmania* promastigotes to induce IL-12 production in human mononuclear cells**. *J. Immunol.* 1997, **159:**2849–2857.
15. Fischer HG, [Nitzgen B](http://www.ncbi.nlm.nih.gov/pubmed?term=%22Nitzgen%20B%22%5BAuthor%5D), [Reichmann G](http://www.ncbi.nlm.nih.gov/pubmed?term=%22Reichmann%20G%22%5BAuthor%5D), [Hadding U](http://www.ncbi.nlm.nih.gov/pubmed?term=%22Hadding%20U%22%5BAuthor%5D): **Cytokine responses induced by *Toxoplasma gondii* in astrocytes and microglial cells.** *Eur. J. Immunol.* 1997, **27:**1539–1548.
16. Kaye PM: **Costimulation and the regulation of antimicrobial immunity.** *Immunol. Today* 1995, **16:**423–427.
17. McMahon-Pratt D, Kima PE, Soong L: **Leishmania amastigote target antigens: the challenge of a stealthy intracellular parasite**. *Parasitol. Today* 1998, **14**:31–34.
