# Supplementary figures and images for: The role of indoleamine 2, 3 dioxygenase in regulating host immunity to leishmania infection
Source: J Biomed Sci. 2012 Jan 9;19(1):5. doi: 10.1186/1423-0127-19-5 (PMC3295648; doi:10.1186/1423-0127-19-5)

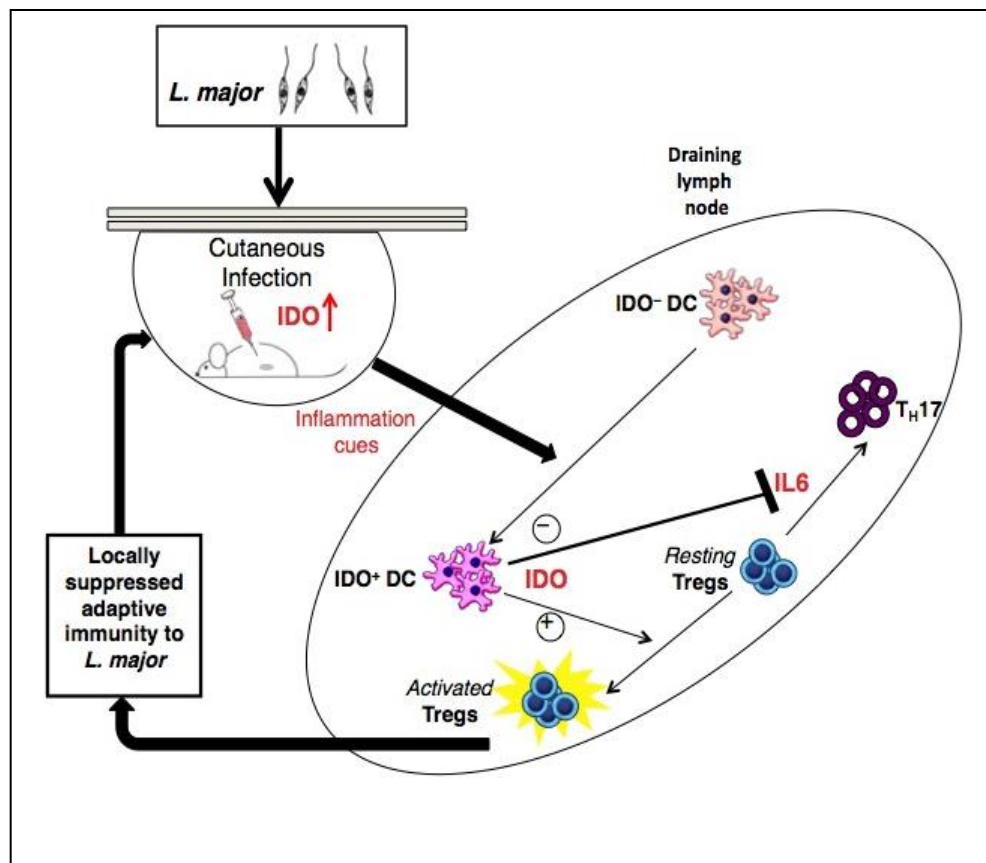

Figure 1 Levi H. C. Makala

Supplement: Additional File 2 — Conceptual model of IDO-mediated activation and effector T cell suppression following L. major infection. The model depicts interactions between IDO+ DCs, Tregs and naïve T cells that drive suppressive and non-suppressive outcomes under IDO-sufficient (+) and IDO-deficient (-) conditions in response to L. major infection. Induced IDO activity in DC's triggers cell stress responses and blocks IL6 production by pDCs themselves, and by other cells (e.g. macrophages), capable of producing IL6. Under conditions of IDO ablation the same stimuli do not create suppression, and instead DCs stimulate naïve T cells, and express IL6, which converts Tregs to TH17 T cells or promotes TH17 differentiation from naïve CD4+ T cells. [file 1423-0127-19-5-S2.PDF]
